# A novel role for Crp in controlling magnetosome biosynthesis in *Magnetospirillum gryphiswaldense* MSR-1

**DOI:** 10.1038/srep21156

**Published:** 2016-02-16

**Authors:** Tong Wen, Fangfang Guo, Yunpeng Zhang, Jiesheng Tian, Ying Li, Jilun Li, Wei Jiang

**Affiliations:** 1 State Key Laboratory of Agro-Biotechnology and Ministry of Agriculture Key Laboratory of Soil Microbiology, College of Biological Sciences, China Agricultural University, Beijing 100193, P. R. China; 2 France-China Bio-Mineralization and Nano-Structures Laboratory, Beijing 100193, P. R. China

## Abstract

Magnetotactic bacteria (MTB) are specialized microorganisms that synthesize intracellular magnetite particles called magnetosomes. Although many studies have focused on the mechanism of magnetosome synthesis, it remains unclear how these structures are formed. Recent reports have suggested that magnetosome formation is energy dependent. To investigate the relationship between magnetosome formation and energy metabolism, a global regulator, named Crp, which mainly controls energy and carbon metabolism in most microorganisms, was genetically disrupted in *Magnetospirillum gryphiswaldense* MSR-1. Compared with the wild-type or complemented strains, the growth, ferromagnetism and intracellular iron content of *crp*-deficient mutant cells were dramatically decreased. Transmission electron microscopy (TEM) showed that magnetosome synthesis was strongly impaired by the disruption of *crp*. Further gene expression profile analysis showed that the disruption of *crp* not only influenced genes related to energy and carbon metabolism, but a series of crucial magnetosome island (MAI) genes were also down regulated. These results indicate that Crp is essential for magnetosome formation in MSR-1. This is the first time to demonstrate that Crp plays an important role in controlling magnetosome biomineralization and provides reliable expression profile data that elucidate the mechanism of Crp regulation of magnetosome formation in MSR-1.

Magnetosomes are intracellular biological membrane-enveloped nano-sized magnetic particles formed by magnetotactic bacteria (MTB)^1^,^2^. Research has suggested that MTB synthesize magnetosomes according to the following steps: invagination of the inner membrane, magnetosome protein localization, alignment of the magnetosomes in the cell, and crystal formation^3^,^4^. It is believed that the magnetosome island (MAI), a large unstable genomic region that spans 80–150 kb in length and is found in many types of MTBs, governs magnetosome synthesis^5^. However, due to its complexity and the fact that various proteins located outside the MAI are also required for magnetosome formation, the precise mechanism of magnetosome formation has not yet been clearly expounded. As MTBs are the simplest model for studying biomineralization, understanding the mechanism of magnetosome synthesis in MTB will lay the groundwork for biomineralization research on magnetosomes-like particles in higher organisms such as bees and pigeons as well as human brains^6^,^7^,^8^.

Recent reports have shown that an ATPase is involved in ferrous ion uptake in *Magnetospirillum magneticum* AMB-1^9^. Additionally, a magnetosome membrane protein, MamK, has been shown to function as both an ATPase and a GTPase^10^. These results suggest that the synthesis of the magnetosome in MTB is an energy-dependent process and that the metabolic energy in the cell influences magnetosome formation.

3′–5′-cyclic adenosine monophosphate (cAMP) receptor protein (Crp) is an important global transcriptional regulator. It was also the first protein to be purified^11^ and crystallized^12^ and is the most well-characterized regulator in *Escherichia coli*^13^. In most species, the main function of Crp is to control energy metabolism, and proteins belonging to this family have a similar regulatory mechanism. First, cAMP binds Crp, inducing a conformational change that activates Crp. Second, Crp binds to the promoter region of a target gene and induces a bend in the DNA. Finally, Crp interacts with RNA polymerase to activate transcription of the target gene^14^,^15^. To investigate the relationship between magnetosome formation and energy metabolism, we disrupted the *crp* gene in *Magnetospirillum gryphiswaldense* MSR-1 and complemented the mutant strain. Phenotypic analysis revealed that the ferromagnetism and intracellular iron content decreased dramatically in the mutant, and magnetosome synthesis was strongly impaired. In addition, the complemented strain phenocopied the wild-type strain. To understand the mechanism of Crp regulation of magnetosome synthesis in *M. gryphiswaldense* MSR-1, transcriptional expression profiles of the MSR-1 wild-type and *crp* mutant strains were compared. The results of high-throughput sequencing of the total mRNA for both the wild-type and *crp* mutant were analyzed and classified through Gene Ontology (GO) functional enrichment^16^ and Kyoto Encyclopedia of Genes and Genomes (KEGG) pathway enrichment^17^; differentially expressed genes were subsequently enriched and classified. The results indicate that many pathways involved in carbon and energy metabolism were affected by the deletion of *crp*, including those involved in pentose and glucuronate interconversions, oxidative phosphorylation and peptidoglycan biosynthesis. In addition, many genes located on the MAI were down regulated by the disruption of *crp*. These results indicated that the global carbon and energy metabolism regulator Crp plays a key role in controlling magnetosome biosynthesis in *M. gryphiswaldense* MSR-1.

## Results

### Construction and identification of the *crp* mutant and its complemented strain

**Figure 1:**
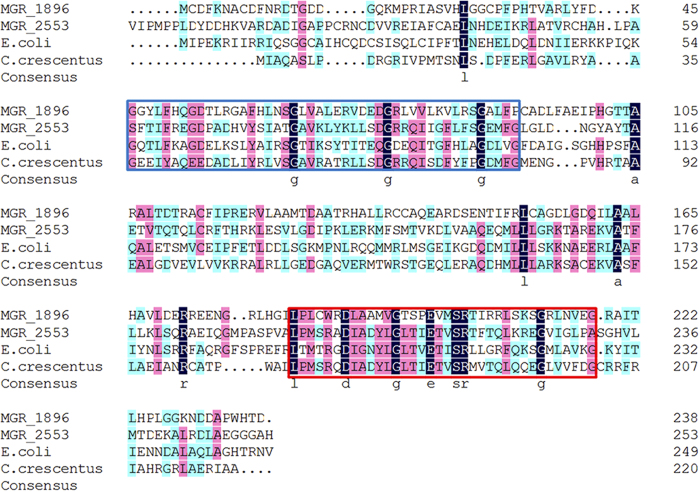
Multiple alignments of Crp/Fnr family transcriptional regulators from MSR-1 (MGR_1896 and MGR_2553), *E. coli* (U068_c0718) and *C. crescentus* (Caul_2975). Conserved amino acid residues were highlighted by navy blue, proposed cyclic nucleotide-binding domain was encircled by blue and helix-turn-helix DNA-binding domain was encircled by red.

In MSR-1, *crp* (MGR_1896) is a 717 bp-long gene that encodes a 238 amino acid residue protein named Crp, which is a predicted transcriptional regulator belongs to Crp/Fnr family. By comparing with the previously reported MgFnr (MGR_2553) of Crp/Fnr family protein in MSR-1^18^, along with two other Crp/Fnr family proteins from *E. coli* (U068_c0718, GenBank: CP011342.2) and *Caulobacter crescentus* (Caul_2975, GenBank: CP000927.1), an alphaproteobacterial model organism which is closely related to MSR-1^19^, a high homology is shown among the four proteins in their cyclic nucleotide-binding domain (Fig. 1 blue frame) and HTH (helix-turn-helix) DNA-binding domain (Fig. 1 red frame). This result suggests the functional similarity among these proteins. In order to determine the function of Crp in MSR-1, a *crp* disruption mutant was constructed by bi-parental conjugation in wild-type MSR-1 (WT). First, the *crp* gene was replaced by a gentamicin (Gm) resistance cassette (Fig. S1A). Then, the resulting mutant, named crp-M, was confirmed by PCR (Fig. S1B). To ensure that the changes in phenotypes seen in crp-M were due to the disruption of *crp*, a complemented strain of crp-M, named crp-C, was constructed by introducing a plasmid with an inducible *crp* cassette into crp-M. Complementation was confirmed by amplifying the *crp* gene fragment (Fig. S1C).

### crp-M has a low intracellular iron content and no magnetism

**Figure 2:**
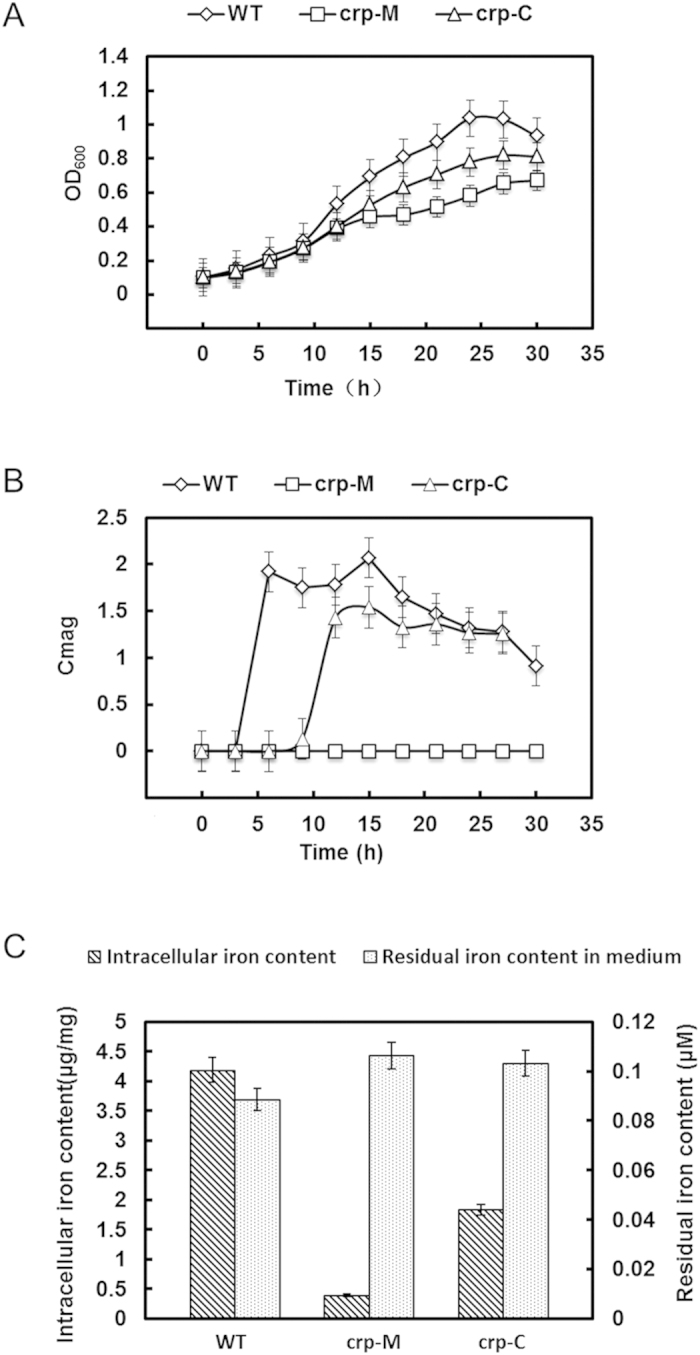
Growth, magnetism, and iron content analysis of various strains. (**A**) Growth measurements of the WT, crp-M and crp-C strains under the same cultivation conditions; (**B**) Cmag measurements of the WT, crp-M and crp-C strains under the same cultivation conditions; (**C**) Residual iron levels in the medium and intracellular iron concentrations detected in cultures with the WT, crp-M and crp-C strains. All experiments were independently repeated three times to ensure their reproducibility.

To investigate the phenotype of crp-M, the growth and Cmag (defined in methods) curves of each strain (WT, crp-M and crp-C) were detected over time. The crp-M strain grew slower than the WT and crp-C strains (Fig. 2A). Interestingly, the crp-M strain showed no magnetism during any growth phase, and the crp-C strain phenocopied the WT when 0.2mM isopropyl-β-d-thiogalactoside (IPTG) was added to the sodium lactate medium (SLM) prior to inoculation, although not fully complemented (Fig. 2B), and it could be due to the unstability or loss of plasmid or low concentration of IPTG. To determine the amount of iron uptake, these three strains were incubated in SLM medium with 60 μM additional ferric citrate. After 24 h of growth, the intracellular iron content of the WT strain (4.18 ± 0.32 μg/mg) and the crp-C strain (1.83 ± 0.35 μg/mg) was approximately 10.45− and 4.58− fold higher, respectively, compared with the crp-M strain (0.40 ± 0.03 μg/mg). Despite these results, there was little difference in the amount of residual iron in the medium between these three strains (Fig. 2C).

### Magnetosome synthesis was strongly impaired in the crp-M strain

**Figure 3:**
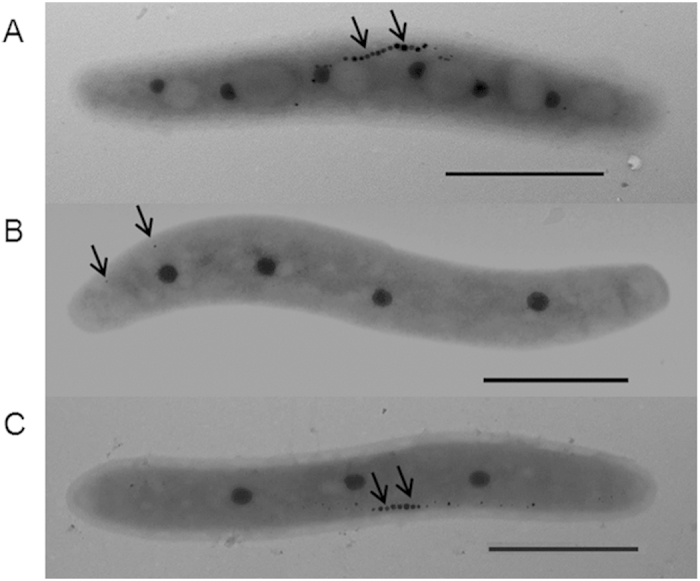
Transmission electron microscopy (TEM) images of the WT (**A**), crp-M (**B**) and crp-C (**C**) strains, magnetosomes or magnetosome chains were denoted by arrows.

To determine why crp-M displayed no magnetism and had a low intracellular iron content, TEM was used to visualize the magnetosomes synthesized in MSR-1. Cells of each strain were cultured under the same condition, and 30 randomly selected cells were used for magnetosome observation by TEM. Morphologically, magnetosomes synthesized in the WT were typically cubo-octahedral in shape, approximately 30–40 nm in diameter and arranged in a line (Fig. 3A). Magnetosomes formed by the complemented strain had a similar shape, size, and arrangement as those formed in the WT strain (Fig. 3C). However, the magnetosome formation was strongly impaired by the disruption of *crp*, and only few defective magnetite nanoparticles were found scattered in the crp-M strain (Fig. 3B). This result demonstrates that the Crp protein is essential for magnetosome formation in MSR-1.

### Illumina sequencing and genome mapping of WT and crp-M

Two RNA-seq libraries were prepared from MSR-1 WT and crp-M for gene expression analysis. The WT and crp-M libraries generated 9.48 and 8.79 million raw reads, respectively. After the analysis of raw data using the NGSQC Toolkit (v2.3), 9.15 and 8.49 million clean reads were obtained, and more than 99% were considered clean at the Q20 level (sequence error probability of 1%). These clean reads were mapped to the *M. gryphiswaldense* MSR-1 genome (MGMSRv2, 4365796 bp), resulting in 9058456 mapped reads for WT and 8440498 mapped reads for crp-M (Table 1).

### Identification of differentially expressed genes (DEGs) and GO enrichment

**Figure 4:**
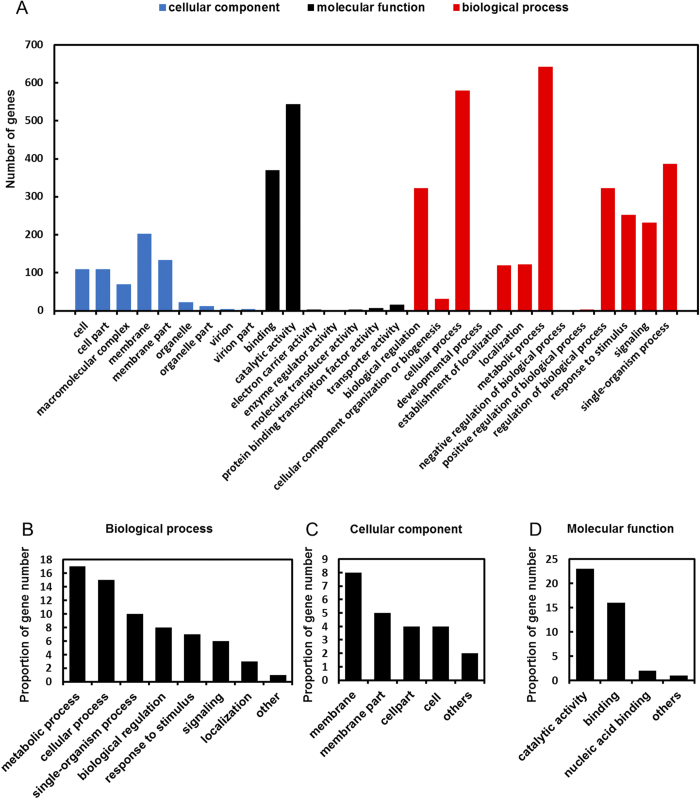
GO classification of genes from the MSR-1 expression profile results (**A**). The results are summarized in three broad categories: biological process (**B**), cellular component (**C**) and molecular function (**D**).

**Figure 5:**
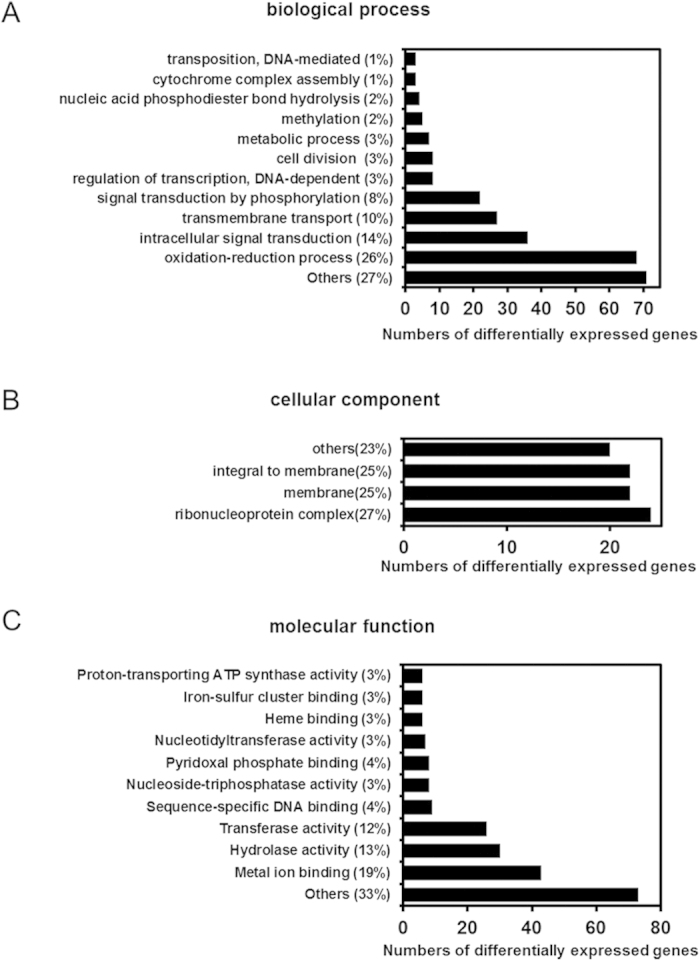
GO classifications of genes differentially expressed between WT and crp-M strains under three categories: biological process (**A**), cellular component (**B**) and molecular function (**C**).

Transcript expression levels were calculated using RPKM (reads per kilobase of exon model per million mapped reads). We defined DEGs as a fold change in the normalized RPKM expression values of at least 2 in either direction of log_2_ ratio ≥ 1. When compared to WT, a total of 356 genes were down regulated and 551 genes were up regulated in crp-M. Gene Ontology classification and enrichment were used to classify the functions of each mapped gene. These classifications are based on the GO classification system (http://geneontology.org/) that classified the genes into various sub-categories according to their common characteristics or functions. A total of 4311 mapped genes were classified using the complete set of GO terms into three broad categories: biological process, cellular component and molecular function (Fig. 4A). In the biological process category, the largest proportion of genes were classified within the metabolic (GO: 0008152) and cellular process (GO: 0009987) sub-categories (Fig. 4B). Under the cellular component category, the largest subset of genes belonged to the membrane (GO: 0016020) and membrane part (GO: 0044425) sub-categories (Fig. 4C). Under the molecular function category, the largest subset of genes belonged to the binding (GO: 0005488) and catalytic activity (GO: 0003824) sub-categories (Fig. 4D). Of the 907 genes differentially expressed between the WT and crp-M, 562 were annotated and classified according to the GO classification rules as shown in Fig. 5. Figure 5A shows that most of the differently expressed genes belong to the oxidation-reduction process (26%), intracellular signal transduction (14%) and transmembrane transport (10%) sub-categories in the biological process category. Under the cellular component sub-category, most of the differently expressed genes were classified as membrane (25%) and membrane related (25%) (Fig. 5B). For the molecular function sub-category, genes involved in metal ion binding (19%), hydrolase activity (13%) and transferase activity (12%) were the most affected in the *crp* mutant (Fig. 5C).

### KEGG pathway enrichment analysis

KEGG pathway enrichment analysis was performed to analyze the metabolic pathways influenced by Crp. The 214 genes that were differentially expressed between WT and crp-M mapped to 8 KEGG pathways (FDR ≤ 0.001) (Table 2). Among these pathways, “Ribosome” contained the highest percentage of genes (6.11%), followed by “Oxidative phosphorylation” (5.38%). Within the Oxidative phosphorylation pathway, genes associated with protein synthesis and energy metabolism were more influenced by Crp than other genes. This is consistent with the known function of Crp in *E. coli*.

### Quantitative real-time reverse transcription polymerase chain reaction (qRT- PCR) analysis

**Figure 6:**
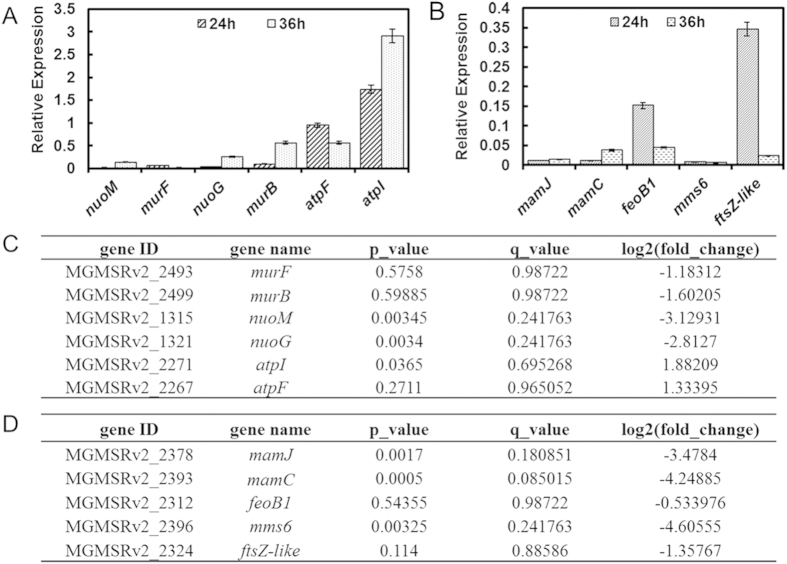
Genes selected for validation by qRT-PCR, samples taken at 24 and 36 h were used for this assay, relative expression level of WT for each gene was set as 1 and not shown in the data. (**A**) expression levels of select genes related to energy or carbon metabolism as determined by qRT-PCR; (**B**) expression level of certain MAI genes that are crucial for magnetosome synthesis as determined by qRT-PCR; (**C**) The expression level of select energy or carbon metabolism genes as determined by RNA-seq; (**D**) The expression levels of select MAI genes as determined by RNA-seq.

To validate the expression profile results, qRT-PCR was used to determine the expression levels of a series of randomly selected genes identified by RNA-seq as being differentially expressed between WT and crp-M. According to the KEGG pathway enrichment analysis, the expression level of the *nuo* (NADH-quinone oxidoreductase components), *atp* (F-type ATPase components) and *mur* (in charge of peptidoglycan biosynthesis) gene operons were the most influenced by *crp* disruption (See Fig. S2 and S3). Therefore, genes located within these clusters were selected for qRT-PCR confirmation. The *rpoA* (encodes RNA polymerase alpha subunit, locus tag MGMSRv2_0062), which is a well-known housekeeping gene and has a stable expression level^20^, was selected as the reference gene. The relative expression level of WT for all genes tested was set to 1. The trends in gene expression levels between WT and crp-M determined by qRT-PCR were in accordance with those obtained from RNA-seq (Fig. 6A,C). Due to the unique presence of MAI genes in MTB, the KEGG pathway enrichment did not categorize these genes. However, the differentially expressed genes identified by RNA-seq suggest that MAI genes are Crp dependent (Table S3). Therefore, to determine whether the genes crucial for magnetosome synthesis were influenced by the disruption of *crp*, qRT-PCR was performed for the genes located in this region in WT and crp-M. Almost all of the genes tested were significantly down regulated, which is in accordance with the RNA-seq results (Fig. 6B,D). Afterward, samples taken at 36 h were also used for qPCR in order to identify whether the expression levels of these DEGs changes in stationary growth phases or not.

## Discussion

Most studies on magnetosome synthesis in MTB have focused on the magnetosome gene island (MAI). However, other studies have indicated that genes located outside the MAI are also involved in magnetosome synthesis, including the *fur* family genes^21^ and some types of ferric reductases^22^. Nearly all of these studies focused on genes related to iron and oxygen metabolism. Some reports suggest that when grown under micro-aerobic conditions, cells cannot obtain sufficient oxygen, which is used as the terminal electron acceptor for cellular respiration, and therefore, they begin to take up large amounts of free iron ions to replace oxygen as the electron acceptor during the respiratory chain electron transfer process^23^,^24^. However, no studies have indicated that there is a relationship between magnetosome formation and energy metabolism. To our knowledge, this is the first report that Crp, a global regulator that controls energy and carbon metabolism, plays a key role in magnetosome formation in MSR-1.

More than 100 promoters have been shown to be activated or repressed by the Crp-cAMP regulatory system in *E. coli*, including the well-known lactose metabolism regulatory system and many other genes and operons related to carbon source catabolism^25^. In addition, genes not involved in carbon metabolism can also be regulated by Crp, including certain types of cold-shock proteins^26^ and proteins involved in antibiotic production^27^. The mechanism of Crp regulation, however, may be different in diverse microorganisms^28^,^29^. Therefore, it is significant and interesting to explore the role of Crp in controlling magnetosome formation in MSR-1.

RNA-seq and qRT-PCR analyses were performed to explore why crp-M fails to synthesize normal magnetosomes and to determine whether the regulatory mechanism of Crp in MSR-1 is similar to that in *E. coli*. The results indicate that in MSR-1, Crp not only participates in energy metabolism, but also involved in the regulation of magnetosome island genes. As is the case in *E. coli*, many genes related to energy and carbon metabolism are regulated by Crp in MSR-1 (Table S3), including NADH-quinone oxidoreductase genes, F-type ATPase synthesis genes and a series of peptidoglycan biosynthesis genes^30^. These findings confirm that in MSR-1, Crp performs a similar function compared to that in *E. coli*. Furthermore, the expression levels of numerous MAI-encoded genes related to magnetosome synthesis are down regulated in a *crp* mutant (Table S3), including *mamJ*, which belongs to the *mamAB* cluster, the major component of the MAI^31^. *mamC*, which is a member of the *mamGFDC* cluster, and the deletion of *mamC* leads to smaller and more irregular magnetosomes^32^. The expression level of *mms6*, which plays a key role in the magnetite crystallization process *in vitro*^33^,^34^, was down regulated according to the qRT-PCR analysis as well as the *ftsZ-like* gene, which belongs to the *mamXY* cluster and has a similar function as the *mamGFDC* cluster^35^. The *feoB1* gene belongs to the *feoAB* cluster was also down regulated according to the results, and disruption of *feoB1* results in a decrease in ferrous ion uptake^36^. All of these results strongly suggest that the global regulator Crp plays a role in contributing to the synthesis of the magnetosome in MSR-1, either directly or indirectly. Furthermore, our results suggest that the magnetosome formation process is energy dependent.

## Methods

### Bacteria and growth conditions

The bacterial strains used in this study are described in Table S1. *M. gryphiswaldense* MSR-1 was cultivated in 50 ml of SLM medium (in a 100 mL serum bottle) at 30 ^o^C, 100 rpm^37^. The medium contained (per liter deionized water) 1.5 g sodium lactate, 0.4 g NH_4_Cl, 0.1 g yeast extract, 0.5 g K_2_HPO_4_, 0.1 g MgSO_4_ • 7 H_2_O, 0.05 g sodium thioglycolate and 5 mL of trace element mixture. Ferric citrate was added as an iron source at a final concentration of 60 μM after the medium was autoclaved. All MSR-1 cells used in this study were cultured for 24 h without a special statement.

### Construction of the *crp* disruption mutant

All primers used in this study are listed in Table S2. The *crp* mutant strain was constructed by homologous recombination. Fragments of the upstream (1102 bp) and downstream (1080 bp) regions of *crp* were amplified using primer pairs crp-uf/crp-ur and crp-df/crp-dr, respectively, and the gentamicin (GM) resistance cassette was digested from a pUC-Gm vector with *Sac*I. All of these fragments were then fused and cloned into the *Hin*dIII and *Bam*HI sites of the pUX19 vector to yield pUX**-**Crp. The pUX**-**Crp plasmid was introduced into donor strain *E. coli* S17-1, and the chromosomal copy of the *crp* gene in MSR-1 was replaced with the Gm cassette through biparental conjugation. Mutants were screened from Gm ^r^ Km ^s^ colonies as previously described^38^. The screened *crp* mutant was confirmed by PCR and named crp-M.

### Construction of *crp* complemented strains

The *crp* gene was cloned from the MSR-1 genome using primers ccrp-f and ccrp-r with *Hin*dIII and *Bam*HI restriction sites (1178 bp). The product was ligated into the pMD18-T simple vector (code D104A; TaKaRa Biotechnology, Dalian, China) for DNA sequencing. The target fragment was then digested with *Bam*HI and *Hin*dIII. The resulting *Bam*HI-*Hin*dIII *crp* fragment was ligated into the *Bam*HI and *Hin*dIII sites of pBBR1MCS-2, which are directly upstream of a *lac* promoter and can be induced by lactose or IPTG, resulting in the plasmid named pBBRcrp. The pBBRcrp plasmid was introduced into MSR-1 crp-M by biparental conjugation. Transconjugants were screened from Gm ^r^, Km ^r^ colonies and further confirmed by PCR.

### Cell growth and magnetic response measurements of various strains

To measure growth and magnetism, all strains were grown synchronously in SLM at 30 °C as described above. The optical density (OD_600_) was measured every 3 h using a UV-visible spectrophotometer (UNICO2100; UNICO Instrument Co., Shanghai, China). Magnetism values (Cmag) were calculated from the measurement of the maximal and minimal scattering intensities at OD_600_^39^, and the cell density and Cmag curves were then constructed.

### Measuring intra- and extracellular (residual iron content in the medium) iron levels

All strains were cultured in SLM (containing 60 μM ferric citrate) at 30 °C under micro-aerobic conditions and harvested by centrifugation. The residual iron content in the medium was measured using the ferrozine assay^40^. To determine the intracellular iron content, pellets were washed three times with 20 mM Tris-HCl buffer containing 4 mM EDTA (pH 7.4). Pellets were then dried to a constant weight at 60 °C, dissolved in 1 mL of nitric acid and digested at 100 °C for 3 h. The iron content was measured using an atomic absorption spectrometer (Optima 5300DV, PerkinElmer, Waltham, MA).

### Transmission electron microscope (TEM) observation

All strains used for TEM observation were cultured in SLM (supplemented with 60 μM ferric citrate) at 30 °C. Then cells were collected by centrifugation. Pellets were rinsed twice with double-distilled H_2_O and then resuspended in double-distilled H_2_O. Suspensions were adsorbed onto copper grids and observed by TEM (JEM-2100, JEOL Ltd., Japan, working at 200 kV).

### RNA sequencing by Illumina HiSeq

Total RNA of each strain was extracted and purified using TRIzol reagent (Tiangen, Beijing, China) according to the manufacturer’s instructions. Subsequently, DNase I (Takara, Shiga, Japan) was used to remove DNA from the extracted total RNA. Each sample was quantified and qualified using an Agilent 2100 Bioanalyzer (Agilent Technologies), and 1 μg of total RNA with a RIN (RNA integrity number) value greater than 7 was used to prepare a library. Single-end index libraries were constructed according to the manufacturer’s protocol (NEB Next^®^ Ultra™ RNA Library Prep Kit for Illumina^®^). Large ribosomal RNA was depleted from the total RNA using the RiboMinus Bacteria Module (Invitrogen), and the ribosomal-depleted mRNA was then fragmented and primed with random primers.

First-strand cDNA was synthesized using ProtoScript II Reverse Transcriptase, and second-strand cDNA was synthesized using the Second Strand Synthesis Enzyme Mix. The double-stranded cDNA, purified using AxyPrep Mag PCR Clean-up (Axygen), was then treated with End Prep Enzyme Mix for end repairing, 5′ phosphorylation and dA-tailing in one reaction, followed by ligation to adaptors with a “T” base overhang. Size selection of adaptor-ligated DNA was then performed using an AxyPrep Mag PCR Clean-up kit (Axygen), and fragments of approximately 400 bp (with an approximate insert size of 250 bp) were recovered. Each sample was then amplified by PCR for 11 cycles, with both primers carrying sequences that could anneal with the flow cell to perform bridge PCR and P7 primer carrying a six-base index allowing for multiplexing. The PCR products were purified using an AxyPrep Mag PCR Clean-up kit (Axygen), validated using an Agilent 2100 Bioanalyzer (Agilent Technologies), and quantified by Qubit and real time PCR (Applied Biosystems). Libraries with different indices were then multiplexed and loaded on an Illumina HiSeq instrument according to manufacturer’s instructions (Illumina, San Diego, CA, USA). Sequencing was performed using a 1 × 50 single-end (SE) configuration; image analysis and base calling were conducted by the HiSeq Control Software (HCS) + OLB + GAPipeline-1.6 (Illumina) on the HiSeq instrument. The sequences were processed and analyzed by GENEWIZ. Co, Ltd.

### Quantitative real-time reverse transcriptase PCR (qRT-PCR)

For qRT-PCR assay, both samples cultured for 24 and 36 h were collected and used for total RNA extraction. cDNA of each strain was synthesized using M-MLV reverse transcriptase (Promega Corp., San Luis Obispo, CA, USA), dNTPs, and random primers (Takara, Shiga, Japan) according to the manufacturer’s instructions, and total RNA was used as the template, RNA were extracted from three paralleled cultures of each strain. qRT-PCR analysis was performed with the cDNA template to determine whether the transcription levels of selected genes match the results of the RNA-seq expression profile. The *rpoA* gene (locus tag MGMSRv2_0062), which encodes the DNA-directed RNA polymerase alpha chain in MSR-1, was used as a positive internal control in the qRT-PCR assay. All qRT-PCR reactions were performed with a LightCycler 480 Instrument II (Roche, South San Francisco, CA, USA). SYBR Green I Master kit (Roche) was used for the amplification. The final reaction volume was 20 μL, with a template cDNA concentration of approximately 20 ng and oligo concentrations of 0.5 μM. The reaction program consisted of a pre-incubation step at 95 °C for 5 min followed by 45 cycles of amplification at 95 °C for 10 s, annealing at 62 °C for 15 s, extension at 72 °C for 15 s, and fluorescence measurement at 95 °C for 5 s and 61 °C for 1 min. For each gene tested by qPCR, 3 replications were performed and their mean C_T_ values were used for final data calculating. The expression level of the genes tested was calculated using the threshold cycle (ΔC_T_) method, which is a variation of the Livak method where ΔC_T_ = C_T_ (reference gene) −C_T_ (target gene).

## Additional Information

**How to cite this article**: Wen, T. *et al.* A novel role for Crp in controlling magnetosome biosynthesis in *Magnetospirillum gryphiswaldense* MSR-1. *Sci. Rep.* **6**, 21156; doi: 10.1038/srep21156 (2016).

## Supplementary Material

Supplementary Information

## Figures and Tables

**Table 1:** Statistical analysis of sequence data and genome mapping.

Samples	WT		crp-M	
	Raw data	Clean data	Raw data	Clean data
Total reads	9480773	9156216	8790500	8497011
Total bases (bp)	483519423	457810800	448315500	424850550
GC content (%)	55.05	54.93	54.83	54.70
N Base ratio (%)	0.00	0.00	0.00	0.00
Q20 Base ratio (%)	98.80	99.52	98.83	99.52
Q30 Base ratio (%)	96.52	97.92	96.57	97.94
Mapped reads	─	9058456	─	8440498
Mapped reads ratio (%)	─	98.9	─	99.3

*N Base ratio: undetermined base ratio

Q20 Base ratio: error probability of 1%

Q30 Base ratio: error probability of 0.1%

**Table 2:** KEGG enrichment results of differentially expressed genes between the crp-M and the WT strain.

Pathway ID	Pathway	DEGs with pathway annotation (214)	All genes with pathway annotation (1667)	p_value	q_value
ko00040	Pentose and glucuronate interconversions	3 (0.73%)	4 (0.24%)	3.58E-03	0.023293734
ko00550	Peptidoglycan biosynthesis	11 (2.69%)	17 (1.02%)	7.47E-05	0.001145059
ko00300	Lysine biosynthesis	8 (1.96%)	14 (0.84%)	1.79E-03	0.015475196
ko00190	Oxidative phosphorylation	22 (5.38%)	44 (2.64%)	5.46E-05	0.001145059
ko04112	Cell cycle - Caulobacter	11 (2.69%)	21 (1.26%)	1.32E-03	0.013699063
ko00471	D-Glutamine and D-glutamate metabolism	3 (0.73%)	4 (0.24%)	3.58E-03	0.023293734
ko00195	Photosynthesis	7 (1.71%)	9 (0.54%)	8.81E-05	0.001145059
ko03010	Ribosome	25 (6.11%)	50 (3.00%)	1.97E-05	0.001024809

## References

[b1] Jogler C. & Schüler D. Genomics, genetics, and cell biology of magnetosome formation. Annu. Rev. Microbiol. 63, 654–672 (2009). 10.1146/annurev.micro.62.081307.162908 19575557

[b2] Komeili A. Molecular mechanisms of compartmentalization and biomineralization in magnetotactic bacteria. FEMS Microbiol. Rev. 36, 232–255 (2012). 22092030 10.1111/j.1574-6976.2011.00315.x PMC3540109

[b3] Bazylinski D. A. & Frankel R. B. Magnetosome formation in prokaryotes. Nat. Rev. Microbiol. 2, 217–230 (2004). 15083157 10.1038/nrmicro842

[b4] Murat D., Quinlan A., Vali H. & Komeili A. Comprehensive genetic dissection of the magnetosome gene island reveals the step-wise assembly of a prokaryotic organelle. Proc. Natl. Acad. Sci. USA 107, 5593–5598 (2010). 20212111 10.1073/pnas.0914439107 PMC2851823

[b5] Grunberg K. *et al.* Biochemical and proteomic analysis of the magnetosome membrane in *Magnetospirillum gryphiswaldense*. Appl. Environ. Microbiol. 70, 1040–1050 (2004). 14766587 10.1128/AEM.70.2.1040-1050.2004 PMC348919

[b6] Gould J. L., Kirschvink J. L. & Deffeyes K. S. Bees have magnetic remanence. Science 201, 1026–1028 (1978). 17743635 10.1126/science.201.4360.1026

[b7] Walcott C., Gould J. L. & Kirschvink J. L. Pigeons have magnets. Science 205, 1027–1029 (1979). 472725 10.1126/science.472725

[b8] Kirschvink J. L., Kobayashi-Kirschvink A. & Woodford B. J. Magnetite biomineralization in the human brain. Proc. Natl. Acad. Sci. USA 89, 7683–7687 (1992). 1502184 10.1073/pnas.89.16.7683 PMC49775

[b9] Suzuki T., Okamura Y. A., Takeyama H. & Matsunaga T. Cytoplasmic ATPase involved in ferrous ion uptake from magnetotactic bacterium *Magnetospirillum magneticum* AMB-1. FEBS Lett . 581, 3443–3448 (2007). 17618623 10.1016/j.febslet.2007.06.047

[b10] Ozyamak E., Kollman J., Agard D. A. & Komeili A. The bacterial actin MamK *in vitro* assembly behavior and filament architecture. J. Biol. Chem. 288, 4265–4277 (2013). 23204522 10.1074/jbc.M112.417030 PMC3567678

[b11] Zubay G., Schwartz D. & Beckwith J. The mechanism of activation of catabolite-sensitive genes. Cold Spring Harb. Symp. Quant. Biol. 35, 433–435 (1970).

[b12] Mckay D. B. & Steitz T. A. Structure of catabolite gene activator protein at 2.9 Å resolution suggests binding to left-handed B-DNA. Nature 290, 744–749 (1981). 6261152 10.1038/290744a0

[b13] Fic E. *et al.* cAMP receptor protein from *Escherichia coli* as a model of signal transduction in proteins – A Review. J. Mol. Microbiol. Biotechnol. 17, 1–11 (2009). 19033675 10.1159/000178014

[b14] Won H. S. *et al.* Structural understanding of the allosteric conformational change of cyclic AMP receptor protein by cyclic AMP binding. Biochemistry 39, 13953–13962 (2000). 11076538 10.1021/bi000012x

[b15] Lawson C. L. *et al.* Catabolite activator protein: DNA binding and transcription activation. Curr. Opin. Struct. Biol. 14, 10–20 (2004). 15102444 10.1016/j.sbi.2004.01.012 PMC2765107

[b16] Xiang L. X., He D., Dong W. R., Zhang Y. W. & Shao J. Z. Deep sequencing-based transcriptome profiling analysis of bacteria-challenged Lateolabrax japonicus reveals insight into the immune-relevant genes in marine fish. BMC Genomics 11, 1–21 (2010). 20707909 10.1186/1471-2164-11-472 PMC3091668

[b17] Shi G. *et al.* RNA-Seq analysis reveals that multiple phytohormone biosynthesis and signal transduction pathways are reprogrammed in curled-cotyledons mutant of soybean [Glycine max (L.) Merr.]. BMC Genomics 15, 383–393 (2014). 24952381 10.1186/1471-2164-15-510 PMC4078243

[b18] Li Y. *et al.* The oxygen sensor MgFnr controls magnetite biomineralization by regulation of denitrification in *Magnetospirillum gryphiswaldense*. BMC Microbiol. 14, 153 (2014). 24915802 10.1186/1471-2180-14-153 PMC4065386

[b19] Leticia B. *et al.* Regulatory Response to Carbon Starvation in *Caulobacter crescentus*. PloS one 6, e18179 (2011). 21494595 10.1371/journal.pone.0018179 PMC3073932

[b20] Ritz M., Garenaux A., Berge M. & Federighi M. Determination of *rpoA* as the most suitable internal control to study stress response in *C. jejuni* by RT-qPCR and application to oxidative stress. J. Microbiol. Methods 76, 196–200 (2009). 19041906 10.1016/j.mimet.2008.10.014

[b21] Qi L. *et al.* Fur in *Magnetospirillum gryphiswaldense* influences magnetosomes formation and directly regulates the genes involved in iron and oxygen metabolism. Plos One 7, e29572 (2012). 22238623 10.1371/journal.pone.0029572 PMC3251581

[b22] Zhang C. *et al.* Two bifunctional enzymes with ferric reduction ability play complementary roles during magnetosome synthesis in *Magnetospirillum gryphiswaldense* MSR-1. J. Bacteriol. 195, 876–885 (2013). 23243303 10.1128/JB.01750-12 PMC3562108

[b23] Blakemore R. P. Magnetotactic bacteria. Annu. Rev. Microbiol. 36, 217–238 (1982). 6128956 10.1146/annurev.mi.36.100182.001245

[b24] Liu Y. *et al.* Large-scale production of magnetosomes by chemostat culture of *Magnetospirillum gryphiswaldense* at high cell density. Microb. Cell Fact. 9, 8687–8692 (2010). 10.1186/1475-2859-9-99 PMC3019156 21144001

[b25] Kolb A., Busby S., Buc H., Garges S. & Adhya S. Transcriptional regulation by cAMP and its receptor protein. Annu. Rev. Biochem. 62, 749–795, (1993). 8394684 10.1146/annurev.bi.62.070193.003533

[b26] Uppal S. & Jawali N. Cyclic AMP receptor protein (CRP) regulates the expression of *cspA, cspB, cspG* and *cspI*, members of *cspA* family, in *Escherichia coli*. Arch. Microbiol. 197, 497–501 (2015). 25637299 10.1007/s00203-015-1085-4

[b27] Gao C., Hindra, Mulder D., Yin C. & Elliot M. A. Crp is a global regulator of antibiotic production in *Streptomyces*. MBio 3, 214104–214104 (2012). 10.1128/mBio.00407-12 PMC3520106 23232715

[b28] Niu W., Kim Y., Tau G., Heyduk T. & Ebright R. H. Transcription activation at class II CAP-dependent promoters: two interactions between CAP and RNA polymerase. Cell 87, 1123–1134 (1996). 8978616 10.1016/s0092-8674(00)81806-1 PMC4430116

[b29] Stapleton M. *et al.* *Mycobacterium tuberculosis* cAMP receptor protein (Rv3676) differs from the *Escherichia coli* paradigm in its cAMP binding and DNA binding properties and transcription activation properties. J. Biol. Chem. 285, 7016–7027 (2010). 20028978 10.1074/jbc.M109.047720 PMC2844151

[b30] Tomohiro S., Nobuyuki F., Kaneyoshi Y. & Akira I. Novel roles of cAMP receptor protein (CRP) in regulation of transport and metabolism of carbon sources. Plos One 6, e20081 (2011). 21673794 10.1371/journal.pone.0020081 PMC3105977

[b31] Kolinko I. *et al.* Biosynthesis of magnetic nanostructures in a foreign organism by transfer of bacterial magnetosome gene clusters. Nat. Nanotechnol. 9, 193–197 (2014). 24561353 10.1038/nnano.2014.13

[b32] Scheffel A., Gärdes A., Grünberg K., Wanner G. & Schüler D. The major magnetosome proteins MamGFDC are not essential for magnetite biomineralization in *Magnetospirillum gryphiswaldense* but regulate the size of magnetosome crystals. J. Bacteriol. 190, 377–386 (2008). 17965152 10.1128/JB.01371-07 PMC2223754

[b33] Atsushi A., John W. & Tadashi M. A novel protein tightly bound to bacterial magnetic particles in *Magnetospirillum magneticum* strain AMB-1. J. Biol. Chem. 278, 8745–8750 (2003). 12496282 10.1074/jbc.M211729200

[b34] Tanaka M., Mazuyama E., Arakaki A. & Matsunaga T. MMS6 protein regulates crystal morphology during nano-sized magnetite biomineralization *in vivo*. J. Biol. Chem. 286, 6386–6392 (2011). 21169637 10.1074/jbc.M110.183434 PMC3057814

[b35] Ding Y. *et al.* Deletion of the *ftsZ-like* gene results in the production of superparamagnetic magnetite magnetosomes in *Magnetospirillum gryphiswaldense*. J. Bacteriol. 192, 1097–1105 (2010). 20023033 10.1128/JB.01292-09 PMC2812952

[b36] Rong C. *et al.* Ferrous iron transport protein B gene (*feoB1*) plays an accessory role in magnetosome formation in *Magnetospirillum gryphiswaldense* strain MSR-1. Res. Microbiol. 159, 530–536 (2008). 18639631 10.1016/j.resmic.2008.06.005

[b37] Guo F. F. *et al.* Magnetosomes eliminate intracellular reactive oxygen species in *Magnetospirillum gryphiswaldense* MSR-1. Environ. Microbiol. 14, (2012). 10.1111/j.1462-2920.2012.02707.x 22360568

[b38] Yang J. *et al.* MamX encoded by the *mamXY* operon is involved in control of magnetosome maturation in *Magnetospirillum gryphiswaldense* MSR-1. BMC Microbiol. 13, 232–241 (2013). 24020498 10.1186/1471-2180-13-203 PMC3847676

[b39] Zhao L., Dan W., Wu L. F. & Tao S. A simple and accurate method for quantification of magnetosomes in magnetotactic bacteria by common spectrophotometer. J. Biochem. Biophys. Methods 70, 377–383 (2007). 17030397 10.1016/j.jbbm.2006.08.010

[b40] Chem A. Ferrozine-a new spectrophotometric reagent for iron. Anal. Chem. 42 (1970).

