# Supplementary material for: A novel role for Crp in controlling magnetosome biosynthesis in Magnetospirillum gryphiswaldense MSR-1
Source: Sci Rep. 2016 Feb 16;6:21156. doi: 10.1038/srep21156 (PMC4754748; doi:10.1038/srep21156)
Supplement: Supplementary Information [file srep21156-s1.pdf]

SUPPORTING INFORMATION

A novel role for Crp in controlling magnetosome biosynthesis in *Magnetospirillum gryphiswaldense* MSR-1

Tong Wen [1, 2, +], Fangfang Guo [1, +], Yunpeng Zhang [1, 2], Jiesheng Tian [1, 2], Ying Li [1, 2], Jilun Li [1, 2], Wei Jiang [1, 2*]

[1]State Key Laboratory of Agro-Biotechnology and Ministry of Agriculture Key Laboratory of Soil Microbiology, College of Biological Sciences, China Agricultural University, Beijing 100193, P. R. China

[2]France-China Bio-Mineralization and Nano-Structures Laboratory, Beijing 100193, P. R. China

[*]Corresponding, Wei Jiang, State Key Laboratory of Agro-Biotechnology and Ministry of Agriculture Key Laboratory of Soil Microbiology, College of Biological Sciences, China Agricultural University, Beijing 100193, P. R. China. Tel: 86-10-62731440. Fax: 86-10-62732012.E-mail: jiangwei01@cau.edu.cn

[+]These authors contributed equally to this work

Table S1. Bacterial strains and plasmids used in this study

**Strains or plasmids**	**Relevant characteristics**	**Source**
**Strains**		
E. coli DH5α	*endA1 hsdR17* [r $^-$ m $^+$] *supE44 thi-1 recA1 gyrA* [NalR] *relA relA1* Δ[*lacZYA-argF*] *U169 deoR* [Ø80Δ (*LacZ*) M15]	Novagen
E. coli DH5α-T-crps	containing *crp*-up arm-pMD18T; Amp ^{r}	this study
E. coli DH5α-T-crpx	containing *crp*-down arm-pMD18T; Amp ^{r}	this study
E. coli DH5α-9TT-crp	containing pPR9TT-crp	this study
E. coli S17-1	*Thi endA recA hsdR* with RP4-2-Tc::Mu-Km::Tn7 integrated in chromosome; Sm ^{r}, Tra $^+$	Novagen
E. coli S17-1-pux-crp	S17-1 containing pUX-19-CS-Gm-CX; Km ^{r}, Amp ^{r}, Gm ^{r}	this study
E. coli S17-1-mcs-crp	S17-1 containing pBBR1MCS-2-crp; Nx ^{r}, Amp ^{r}, Km ^{r}	this study
E. coli BL21(DE3)	F$^-$*ompT hdS$_B$* (rB$^-$ mB$^-$) *gal dcm* (DE3), general purpose expression host	Novagen
E. coli BL21-Crp	BL21 containing pET28a-crp; Km ^{r}	this study
MSR-1 WT	Wild-type *Magnetospirillum gryphiswaldense*; Nx ^{r}	DSM6361
MSR-1 crp-M	*crp*-deficient mutant containing pBBRMCS-2; Nx ^{r}, Gm ^{r}, Amp ^{r}, Km ^{r}	this study
MSR-1crp-C	The *crp* complemented strains of crp-M	this study
**Plasmids**		
pMD18-T	Linearized vector with T-overhang for direct cloning of PCR fragments with A-overhang, *lacZa*, ori, f1-origin, Amp ^{r}	TaKaRa
pMD18-crp	pMD18 carrying fragment of *crp* gene; Amp ^{r}	this study
pMD18-crps	pMD18 carrying fragment of *crp*-up arm gene; Amp ^{r}	this study
pMD18-crpx	pMD18 carrying fragment of *crp*-down arm gene; Amp ^{r}	this study
pET-28a(+)	Expression vector for *E. coli*, N-terminal 6×His-tag, Km ^{r}	Novagen
pET-28a-crp	Expression of the His-tag-N-terminal protein of Crp; Km ^{r}	this study
pUCGm	pUC19 derivative containing aacC1 (encoding Gm^r gene) Amp ^{r}, Gm ^{r}	-
pUX-19	Suicide vector for MSR-1; Km ^{r}, Amp ^{r}	Novagen
pUXCrp	pUX-19 derivative, carrying *crp* flanking sequences and Gm ^{r} gene; Km ^{r}, Amp ^{r}, Gm ^{r}	this study
pBBR1MCS-2	Broad-host range *lacZ* promoter probe vector; Amp ^{r}, Km ^{r}	this study
pBBCrp	pBBR1MCS-2 carrying fragment of *crp* gene; Amp ^{r}, Km ^{r}	this study

Table S2. Primers used in this study.

**Name**	**Sequence**	**Description**
crp-uf	AAGCTTGCAATCC GCCCTATATCCCTG (*Hind* III)	The 5′ flank sequence of *crp*
crp-ur	GAGCTCGGGGTTCACGCGGCTC (*Sac* I)	The 5′ flank sequence of *crp*
crp-df	GAGCTCGCACATGATT TCTCTCCTGGTC (*Sac* I)	The 3′flank sequence of *crp*
crp-dr	GGATCCGATGAACAAGATGACGTCGCC (*BamH*I)	The 3′flank sequence of *crp*
ccrp-f	AAGCTTCGAAGGTCAATCCCAACAGG (*Hind*III)	Complementation of the Δ*crp*
ccrp-r	GGATCCGCCGACCAAGCCTAAATGTT (*BamH*I)	Complementation of the Δ*crp*
CrpP-f	GGATCCATGTGCGATTTCAAGAATGCAT (*BamH*I)	Expression of Crp protein
CrpP-r	AAGCTTTTATCCGTGTGCCAGGGC (*Hind*III)	Expression of Crp protein
**Primers for quantitative real-time PCR**		
qmamJ-f	TTGATCGCTAATCCCGCAC	qPCR for *mamJ* gene
qmamJ-r	CGGTGATCTTGTTAGGCTGG	qPCR for *mamJ* gene
qmamC-f	CTTTCAACTTGCGCCGTAC	qPCR for *mamC* gene
qmamC-r	CCGGTTATCTGCTTGTCCTTC	qPCR for *mamC* gene
qmms6-f	GCTCATCCTCGGTGTCG	qPCR for *mms6* gene
qmms6-r	TGTGCCGCTTCGATATCAC	qPCR for *mms6* gene
qfeoB1-f	TCTTTTCCAGCTTCTCCTGC	qPCR for *feoB1* gene
qfeoB1-r	CGCGATATCACCTATTCCCAG	qPCR for *feoB1* gene
qftsZ-f	TTATGAGCGAGATGGGCAAG	qPCR for *ftsZ-like* gene
qftsZ-r	TTGATCAGCACTCCCTTGG	qPCR for *ftsZ-like* gene
qnuoM-f	TCATTCTGGGCTATTTCGACG	qPCR for *nuoM* gene
qnuoM-r	GATGGGAGTGAGGAAAGTGG	qPCR for *nuoM* gene
qnuoG-f	GTTGCATCCGCTTCATTTCC	qPCR for *nuoG* gene
qnuoG-r	GATCAAATTGCCCGACAGTTC	qPCR for *nuoG* gene
qmurB-f	ATCCACCTTCGCCAATCC	qPCR for *murB* gene
qmurB-r	AGTGCTTTTCCGATACCTGG	qPCR for *murB* gene
qmurF-f	GTTGATCCCGTTGACCAAAATG	qPCR for *murF* gene
qmurF-r	CGACGCCCAGGAAGATTTC	qPCR for *murF* gene
qatpF-f	TGCCGTTCTATGCCGATG	qPCR for *atpF* gene
qatpF-r	GGCGAGCCTTGATCTTGG	qPCR for *atpF* gene
qatpI-f	CGCAATCCGTCTCAGGG	qPCR for *atpI* gene
qatpI-r	GCAGAAACAGCACCATCAAC	qPCR for *atpI* gene

Table S3. Differentially expressed genes of crp-M compared with WT selected from the expression profile data.

Gene ID	Gene description	Fold change	Regulation	P-value
**Peptidoglycan biosynthesis**				
MGMSRv2_2499	UDP-N-acetylenolpyruvoylglucosamine reductase	-1.6021	down	0.59885
MGMSRv2_2498	UDP-N-acetyl-muramate:alanine ligase	-1.2894	down	0.5068
MGMSRv2_2497	UDP-N-acetylglucosamine:N-acetylmuramyl-(pentapeptide) pyrophosphoryl-undecaprenol N-acetylglucosamine transferase	-1.1122	down	0.67855
MGMSRv2_2495	UDP-N-acetylmuramoylalanine-D-glutamate ligase	-1.0077	down	0.7069
MGMSRv2_2494	UDP-N-acetylmuramyl-tripeptide synthetase	-1.7611	down	0.0488
MGMSRv2_2493	UDP-N-acetylmuramoyl-tripeptide--D-alanyl-D-alanine ligase	-1.1831	down	0.5758
MGMSRv2_2492	UDP-N-acetylmuramyl-tripeptide synthetase	-1.1772	down	0.6546
**NADH-quinone oxidoreductase**				
MGMSRv2_1326	NADH-quinone oxidoreductase subunit B	-2.59423	down	0.0472
MGMSRv2_1324	NADH-quinone oxidoreductase subunit D	-2.23735	down	0.1663
MGMSRv2_1322	NADH-quinone oxidoreductase chain 1	-1.77242	down	0.23175
MGMSRv2_1321	NADH-quinone oxidoreductase subunit G	-2.8127	down	0.0034
MGMSRv2_1316	NADH-ubiquinone oxidoreductase, subunit L	-3.03891	down	0.00285
MGMSRv2_1315	NADH-ubiquinone oxidoreductase, subunit M	-3.12931	down	0.00345
MGMSRv2_1314	NADH-quinone oxidoreductase subunit N	-2.66929	down	0.00445
**ATP synthase**				
MGMSRv2_2267	ATP synthase subunit B	1.33395	up	0.2711
MGMSRv2_2268	ATP synthase subunit B'	1.17761	up	0.3707
MGMSRv2_2269	ATP synthase subunit C	1.39559	up	0.10875
MGMSRv2_2271	ATP synthase protein I	1.88209	up	0.0365
**Magnetosome island genes**				
MGMSRv2_2324	FtsZ-like protein	-1.35767	down	0.114
MGMSRv2_2370	Magnetosome protein MamQ	-3.52374	down	0.01475
MGMSRv2_2373	Magnetosome protein MamO	-4.45103	down	0.0001
MGMSRv2_2378	MamJ protein	-3.4784	down	0.0017
MGMSRv2_2379	Magnetosome protein MamE	-4.00548	down	0.0003
MGMSRv2_2393	Magnetosome protein MamC	-4.51867	down	0.0023
MGMSRv2_2394	Magnetosome protein MamD	-4.24885	down	0.0005
MGMSRv2_2396	Magnetosome protein Mms6	-4.60555	down	0.00325

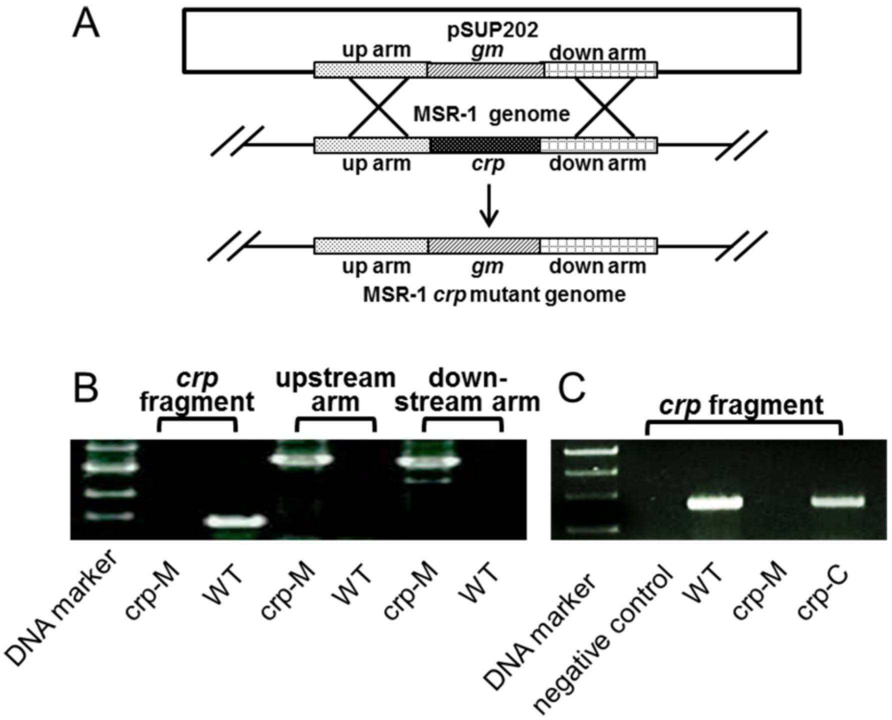

Figure S1. Construction and confirmation of the *crp* mutant (crp-M) and complementary strain (crp-C).

A: Schematic representation of the homologous recombination process; B: confirmation of the *crp* mutation by amplification of the *crp* gene and its up and downstream arms in both the wild type (WT) and crp-M. DNA marker, from top to bottom: 4500 bp, 3000 bp, 2000 bp, 1200 bp, 800 bp and 500 bp; C: confirmation of the crp-C strain, double-distilled H_2O was used as the negative control. DNA marker, from top to bottom: 1200 bp, 800 bp, 500 bp and 200 bp.

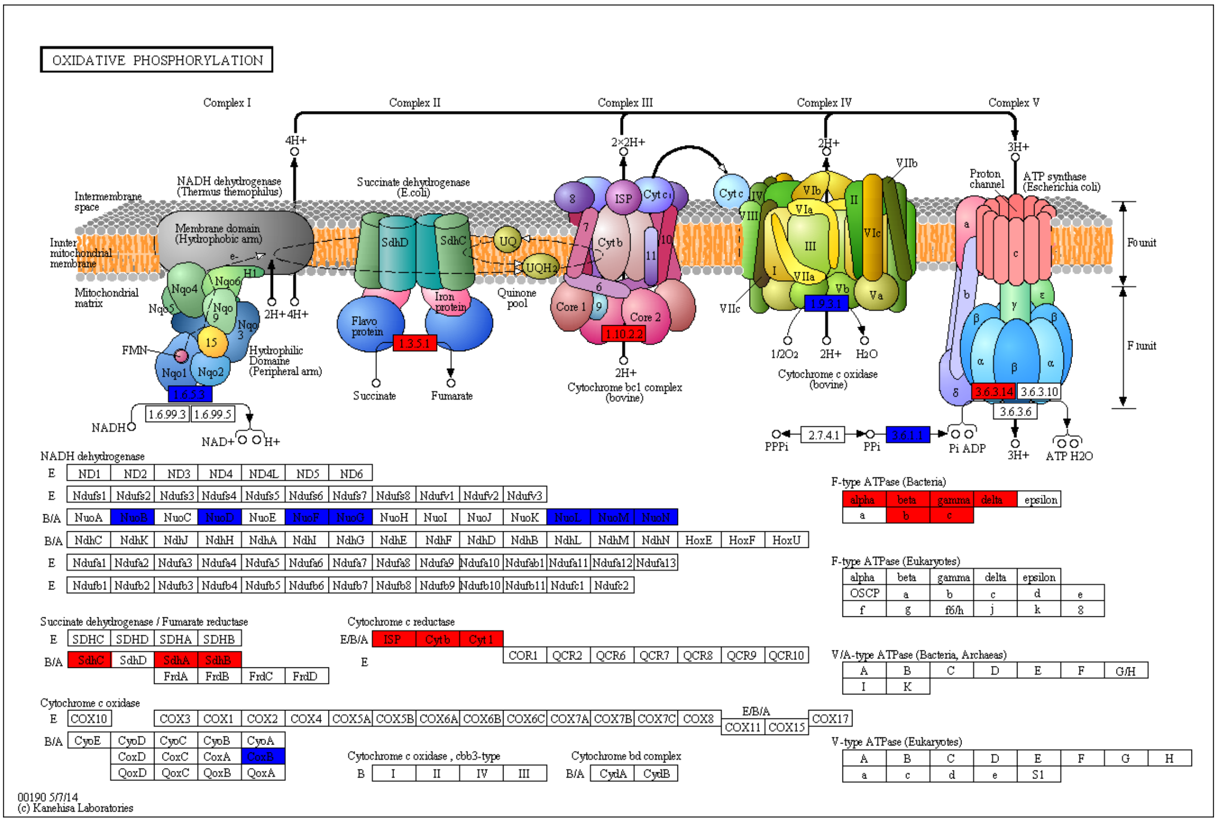

Figure S2. KEGG pathway enrichment of the oxidative phosphorylation pathway. Down regulated pathways in crp-M are indicated in blue, and up regulated pathways in crp-M are indicated in red.

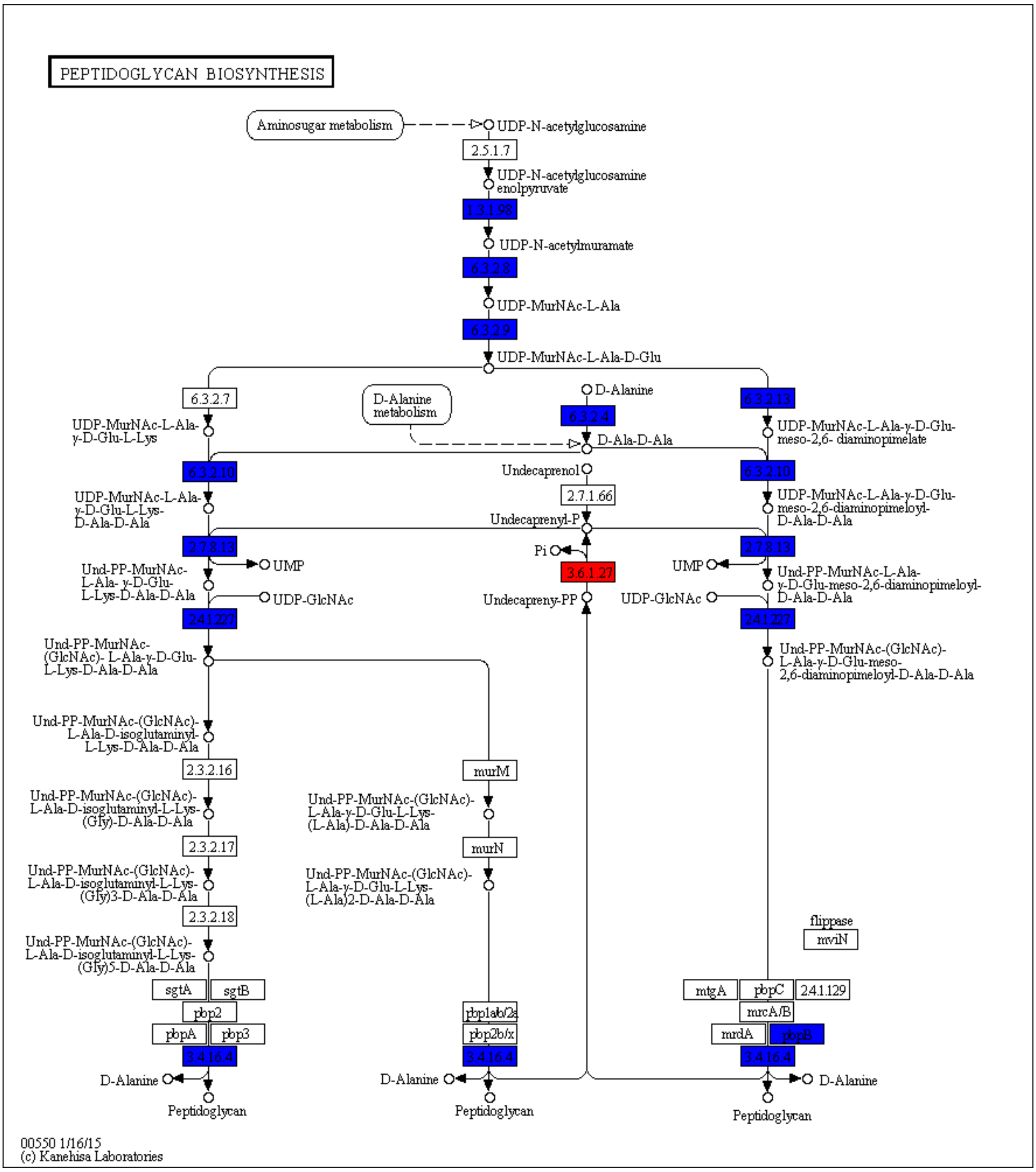

Figure S3. KEGG pathway enrichment of the peptidoglycan biosynthesis pathway. Downregulated pathways in crp-M are indicated in blue, and upregulated pathways in crp-M are indicated in red.
